# Comparação de hastes intramedulares com bloqueios cefálicos e parafuso deslizante DHS no tratamento cirúrgico das fraturas transtrocanterianas instáveis em adultos – Uma revisão sistemática com metanálise

**DOI:** 10.1055/s-0045-1812024

**Published:** 2025-11-18

**Authors:** João Protásio, Paulo Victor Dias Almeida, Mariana Garcia Martins Castro

**Affiliations:** 1 Hospital Geral de Palmas, Universidade Federal do Tocantins, Palmas, TO, Brasil; 2 Secretaria Municipal de Saúde de Palmas, TO, Brasil

**Keywords:** fraturas do quadril, fraturas ósseas, haste intramedular, metanálise, pinos ortopédicos, bone nails, fractures, bone, hip fractures, intramedullary nailing, meta-analysis

## Abstract

**Objetivo:**

Comparar os desfechos clínicos, funcionais e de segurança entre o uso de hastes intramedulares com bloqueios cefálicos ( *cephalomedullary nails* , CMNs, em inglês) e o de parafuso deslizante *dynamic hip screw* (DHS) no tratamento de fraturas transtrocanterianas instáveis (dos graus 31-A2/A3, segundo a classificação da Arbeitsgemeinschaft für Osteosynthesefragen [AO, Associação para o Estudo da Fixação Interna]/Orthopaedic Trauma Association [OTA, Associação de Trauma Ortopédico]), e III–V segundo a classificação de Tronzo).

**Métodos:**

Conduzimos uma revisão sistemática conforme a declaração Preferred Reporting Items for Systematic reviews and Meta-Analyses (PRISMA) de 2020, com buscas nas bases de dados PubMed, Scopus, Embase, Cochrane e Web of Science. Foram incluídos estudos comparativos (ensaios clínicos randomizados e controlados e coortes prospectivas) publicados entre 2000 e 2025. Os desfechos primários foram mortalidade, falha do implante, reoperação e pontuação funcional. Realizamos também uma análise estatística por modelo de efeitos aleatórios, com índice de heterogeneidade (I ^2^ ) e avaliação de qualidade de acordo com a abordagem Grading of Recommendations, Assessment, Development and Evaluation (GRADE).

**Resultados:**

Foram incluídos 18 estudos na metanálise. O grupo CMN apresentou menor tempo cirúrgico (diferença média [DM] = −12,3 minutos), menor sangramento intraoperatório (DM = −88 mL), menor risco de falha mecânica (razão de chances [RC] = 0,42) e menor necessidade de reoperação (RC = 0,58). As pontuações funcionais e a mortalidade não apresentaram diferenças significativas. A qualidade da evidência foi considerada alta para o tempo cirúrgico e as complicações mecânicas, moderada para a reoperação, e baixa para a pontuação funcional.

**Conclusão:**

As CMNs mostraram-se superiores ao DHS no tratamento das fraturas transtrocanterianas instáveis em diversos desfechos clínicos, e devem ser consideradas o tratamento de escolha, especialmente em casos de maior instabilidade. Entretanto, em contextos com limitação de recursos, como o dos hospitais públicos brasileiros, o DHS permanece uma alternativa viável quando utilizado com critério técnico adequado.

## Abstract (en)

**Objective:**

To compare the clinical, functional, and safety outcomes of intramedullary nails with cephalic screws (cephalomedullary nails, CMNs) and the dynamic hip screws (DHSs) in the treatment of unstable intertrochanteric femur fractures (of grades 31-A2/A3 according to the Arbeitsgemeinschaft für Osteosynthesefragen [AO, Association for the Study of Internal Fixation]/Orthopaedic Trauma Association [OTA] and III–V according to the Tronzo classification).

**Methods:**

The present systematic review followed the 2020 guidelines of the Preferred Reporting Items for Systematic reviews and Meta-Analyses (PRISMA) statement, with searches on the PubMed, Scopus, Embase, Cochrane, and Web of Science databases. We included comparative studies (randomized controlled trials or prospective cohorts) published from 2000 to 2025. The outcomes included mortality, implant failure, reoperation, and functional scores. We also conducted an analysis through the random effects model, heterogeneity assessment per I ^2^ , and evidence quality per the Grading of Recommendations, Assessment, Development and Evaluation (GRADE) approach.

**Results:**

A total of 18 studies were included. The CMN group presented shorter operative time (mean difference [MD] = −12.3 minutes), lower intraoperative bleeding (MD = −88 mL), reduced mechanical failure (odds ratio [OR] = 0.42), and fewer reoperations (OR = 0.58). There were no significant differences regarding the functional scores and mortality. The quality of the evidence was high for operative time and mechanical complications, moderate for reoperation, and low for the functional scores.

**Conclusion:**

The CMNs were superior to DHS in multiple clinical outcomes for unstable trochanteric fractures, and they should be considered the treatment of choice, especially in cases of greater instability. However, in resource-limited settings, such as those of the Brazilian public hospitals, DHS remains a viable alternative when used with appropriate technical criteria.

## Introdução

As fraturas transtrocanterianas do fêmur proximal representam um problema importante de saúde pública global, pois são uma das principais causas de hospitalização ortopédica em idosos. 1 Estima-se que sua incidência aumente exponencialmente nas próximas décadas devido ao envelhecimento populacional, com previsão de 4,5 milhões de casos anuais até 2050. 2 Essas fraturas estão associadas a elevadas taxas de morbidade e mortalidade, com impacto direto sobre custos hospitalares e sobrecarga dos sistemas de saúde. 3

A escolha do método de fixação para fraturas transtrocanterianas permanece objeto de debate na literatura ortopédica atual, especialmente quando se consideram as fraturas instáveis. 4 Estas, classificadas como de graus III a V na classificação de Tronzo, ou 31-A2/A3, segundo a classificação da Arbeitsgemeinschaft für Osteosynthesefragen (AO, Associação para o Estudo da Fixação Interna, em alemão/Orthopaedic Trauma Association [OTA, Associação de Trauma Ortopédico, em inglês]), caracterizam-se pela cominuição posteromedial, traço reverso ou extensão subtrocantérica, e apresentam maior risco de colapso, consolidação viciosa e falha de implante. 5

Tradicionalmente, o sistema extramedular conhecido como *dynamic hip screw* (DHS) foi amplamente utilizado no manejo dessas fraturas. 6 Este implante apresenta vantagens como relativa facilidade técnica, menor curva de aprendizado e ampla disponibilidade em serviços de menor complexidade. No entanto, seu posicionamento lateral exerce maior força de tensão sobre o implante, o que pode comprometer a estabilidade em fraturas com maior instabilidade. 7

Nas últimas duas décadas, as hastes intramedulares com bloqueio cefálico ( *cephalomedullary nails* [CMNs], como a *proximal femoral nail antirotation* [PFNA], *Gamma nail* e Intertan) têm ganhado destaque por apresentarem vantagens biomecânicas significativas. 8 9 Por seu posicionamento intramedular, estes implantes proporcionam braço de alavanca reduzido, melhor distribuição de cargas axiais, menor momento flexor e superior controle rotacional do fragmento proximal. 10 Estudos biomecânicos 11 12 demonstraram que estas propriedades são particularmente vantajosas em fraturas com instabilidade posteromedial ou traço reverso.

A literatura recente contém diversos estudos que comparam esses dois métodos de fixação, mas com resultados por vezes conflitantes. Ao passo que alguns estudos 13 14 demonstram superioridade das CMNs em termos de complicações mecânicas, tempo cirúrgico e perda sanguínea, outros trabalhos 15 16 não encontraram diferenças estatisticamente significativas em desfechos como pontuação funcional, taxa de reoperação ou mortalidade.

Adicionalmente, fatores como custo do implante, disponibilidade em diferentes contextos socioeconômicos e experiência do cirurgião são variáveis que frequentemente influenciam a decisão terapêutica, mas raramente são abordadas de maneira sistemática nas publicações. 17 18 No cenário brasileiro, em que coexistem diferentes realidades assistenciais, a escolha do implante frequentemente não se baseia somente em evidências científicas: se baseia também em disponibilidade material e familiaridade técnica. 19

Diante desse cenário, torna-se fundamental uma análise abrangente e atualizada das evidências disponíveis que compare estes dois métodos de fixação especificamente para fraturas transtrocanterianas instáveis. Esta revisão sistemática com meta-análise tem como objetivo comparar os principais desfechos clínicos e funcionais das CMNs e do DHS no tratamento das fraturas transtrocanterianas instáveis em adultos, a fim de oferecer evidência científica sólida que auxilie na tomada de decisão cirúrgica.

## Materiais e Métodos

**Fig. 1:**
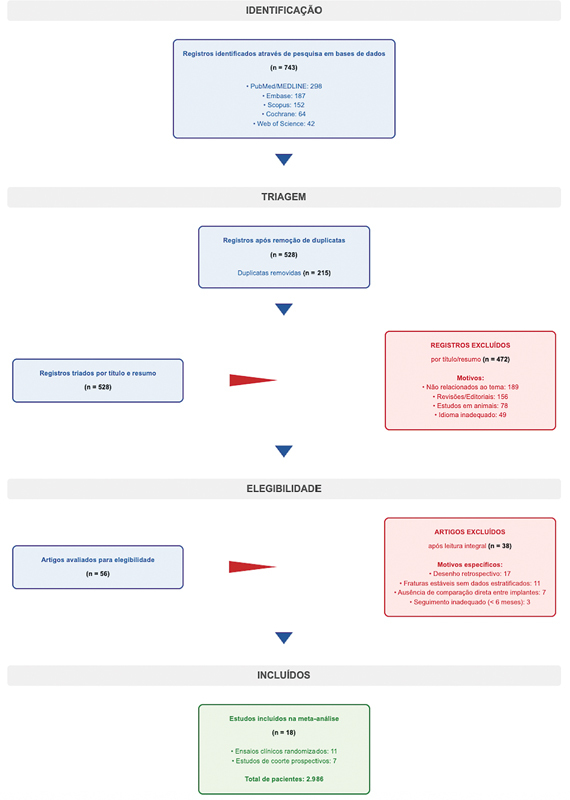
Fluxograma da declaração Preferred Reporting Items for Systematic reviews and Meta-Analyses (PRISMA) da seleção de estudos para inclusão na revisão sistemática e metanálise.

Este estudo foi conduzido conforme as diretrizes de 2020 da declaração Preferred Reporting Items for Systematic Reviews and Meta-Analyses (PRISMA), como ilustrado na Fig. 1 . O protocolo desta revisão não foi registrado prospectivamente em uma plataforma como a International Prospective Register of Systematic Reviews (PROSPERO), o que constitui uma limitação metodológica reconhecida pelos autores. Esta decisão deveu-se ao caráter retrospectivo do planejamento do estudo, iniciado após a coleta preliminar de dados. A estratégia de pesquisa foi elaborada utilizando o modelo Population, Intervention, Comparison, Outcomes, and Study (PICOS, População, Intervenção, Comparação, Desfechos e Estudo):

**População:** adultos (≥ 18 anos) com fraturas transtrocanterianas instáveis do fêmur (classificadas como Tronzo III–V ou AO/OTA 31-A2/A3).
**Intervenção:** fixação com CMNs (incluindo *Gamma nail* , PFNA, Intertan, *trochanteric fixation nail* [TFN], e outros modelos).
**Comparação:** DHS com ou sem placa estabilizadora trocantérica.
**Desfechos:** primários – mortalidade (em 30 dias e 1 ano), falha do implante ( *cut-out* , *cut-through* , quebra do implante), necessidade de reoperação e pontuação funcional (segundo o Harris Hip Score [HHS] e o Parker-Palmer Mobility Score); e secundários: tempo cirúrgico, perda sanguínea estimada, tempo de internação, tempo para consolidação, infecção de sítio cirúrgico, eventos tromboembólicos e dor persistente.
**Tipo de estudo:** ensaios clínicos randomizados e controlados (ECRCs) e estudos de coorte prospectivos.

A inclusão de estudos de coorte prospectivos justifica-se pelo número limitado de ECRCs que avaliam especificamente fraturas transtrocanterianas instáveis. Uma busca preliminar identificou apenas 5 ECRCs focados exclusivamente em fraturas instáveis nos últimos 10 anos, número insuficiente para uma meta-análise robusta. A inclusão de coortes prospectivas de alta qualidade permite aumentar o poder estatístico mantendo adequado rigor metodológico, conforme recomendado pelo *Cochrane Handbook* para situações com escassez de ECRCs.

### Estratégia de busca

Foi realizada pesquisa abrangente nas seguintes bases de dados eletrônicas: PubMed/MEDLINE, Embase, Scopus, Cochrane Library (CENTRAL) e Web of Science. A busca incluiu estudos publicados entre janeiro de 2000 e janeiro de 2025, sem restrição de idioma. Foram utilizados os seguintes termos e suas combinações: *intertrochanteric fracture* , *pertrochanteric fracture* , *trochanteric fracture* , *unstable fracture* , *reverse obliquity* , *intramedullary nail* , *cephalomedullary nail* , *Gamma nail* , *PFNA* , *Intertan* , *dynamic hip screw* , *DHS* e *extramedullary fixation* . A estratégia de busca completa para cada base de dados está disponível no material suplementar.

Adicionalmente, foram verificadas as referências bibliográficas dos estudos incluídos e de revisões sistemáticas anteriores para a identificação de estudos potencialmente relevantes não capturados pela busca eletrônica. Também foram consultados registros de ensaios clínicos (ClinicalTrials.gov, World Health Organization [WHO] International Clinical Trials Registry Platform [ICTRP]) para a identificação de estudos em andamento ou não publicados.

### Critérios de elegibilidade

Foram incluídos estudos que atenderam aos seguintes critérios: 1) ECRCs ou estudos de coorte prospectivos; 2) participantes adultos (≥ 18 anos) com fraturas transtrocanterianas instáveis (AO/OTA 31-A2/A3 ou Tronzo III–V); 3) comparação direta entre CMNs e DHS; 4) avaliação de pelo menos 1 dos desfechos primários; e 5) seguimento mínimo de 6 meses.

Foram excluídos: 1) estudos observacionais retrospectivos; 2) relatos de caso, séries de casos e revisões narrativas; 3) estudos que incluíam fraturas estáveis (AO/OTA 31-A1 ou Tronzo I–II) sem dados estratificados para fraturas instáveis; 4) estudos com foco exclusivo em populações específicas (como pacientes com fratura patológica); e 5) estudos com publicações duplicadas ou com sobreposição de populações.

### Seleção dos estudos e extração de dados

A triagem dos títulos e resumos foi realizada de forma independente por dois revisores, ambos com experiência em revisões sistemáticas (mínimo de três revisões publicadas) e treinamento formal na metodologia PRISMA e em ferramentas de avaliação de qualidade por meio do Cochrane Training. Os revisores participaram de sessão de calibração com 50 resumos antes do início da triagem formal. Os estudos considerados potencialmente elegíveis foram selecionados para leitura integral. Discordâncias foram resolvidas por consenso ou com a participação de um terceiro revisor. Todo o processo de seleção está ilustrado na Fig. 1 .

A extração de dados foi realizada de forma independente, utilizando um formulário padronizado que incluiu: 1) características do estudo (autor, ano, país, desenho, período de recrutamento e tamanho amostral); 2) características da população (idade, sexo, classificação da fratura e comorbidades); 3) características da intervenção (tipo específico de implante e técnica cirúrgica); 4) características da comparação (técnica cirúrgica e uso de placa trocantérica adicional); 5) desfechos avaliados; 6) tempo de seguimento; e 7) resultados para cada desfecho.

### Avaliação da Qualidade Metodológica

A qualidade metodológica dos estudos incluídos foi avaliada por dois revisores independentes. Para os ECRCs, utilizou-se a ferramenta de risco de viés da Cochrane (RoB 2.0), que analisa 5 domínios: 1) processo de randomização – avaliação da geração da sequência e da ocultação da alocação; 2) desvios da intervenção pretendida – consideração do cegamento de participantes e profissionais; 3) dados ausentes – análise da perda de seguimento e das exclusões; 4) mensuração dos desfechos – verificação do cegamento dos avaliadores; e 5) seleção do resultado relatado – comparação do protocolo e da publicação. Para os estudos de coorte prospectivos, foi utilizada a escala de Newcastle-Ottawa, que avalia 3 domínios: seleção da população (4 itens: representatividade da coorte exposta, seleção da coorte não exposta, verificação da exposição e demonstração de que o desfecho não estava presente no início); comparabilidade dos grupos (1 item: controle de fatores de confusão); e avaliação do desfecho (3 itens: avaliação independente, seguimento adequado e perdas de seguimento), com pontuação máxima de 9 estrelas. Estudos com ≥ 7 estrelas foram considerados de boa qualidade metodológica.

A qualidade geral da evidência para cada desfecho foi avaliada utilizando a metodologia Grading of Recommendations, Assessment, Development and Evaluation (GRADE), considerando os seguintes critérios: risco de viés, inconsistência, imprecisão, evidência indireta e viés de publicação.

### Análise Estatística

Os dados foram analisados por meio do programa Review Manager (RevMan, The Cochrane Collaboration), versão 5.4. Para desfechos dicotômicos (mortalidade, falha do implante, reoperação), calculou-se a razão de chances (RC) com IC 95%. Para desfechos contínuos (tempo cirúrgico, perda sanguínea e pontuação funcional), utilizou-se a diferença média (DM) com IC 95%.

Considerando as heterogeneidades clínica e metodológica esperadas entre os estudos, optou-se por um modelo de efeitos aleatórios para todas as análises. A heterogeneidade estatística foi avaliada mediante o teste do qui-quadrado (χ ^2^ ) e quantificada pelo índice I ^2^ , sendo classificada como baixa (I ^2^ < 25%), moderada (I ^2^ entre 25% e 75%) ou alta (I ^2^ > 75%). Para explorar potenciais fontes de heterogeneidade, foram realizadas análises de sensibilidade (excluindo estudos com alto risco de viés) e análises de subgrupos (por tipo específico de CMN, por classificação da fratura e por uso de placa trocantérica adicional no grupo DHS). Uma análise de sensibilidade também foi conduzida, na qual foram separados ECRCs de estudos de coorte, para confirmar a robustez dos resultados principais.

## Resultados

### Seleção dos estudos

Com a busca sistemática, foram identificados 743 registros, 215 dos quais eram duplicatas e foram removidos. Após triagem dos 528 títulos e resumos restantes, 56 artigos foram selecionados para a leitura integral. Os motivos para a exclusão dos 472 registros na fase de triagem foram: artigos não relacionados ao tema (n = 189), artigos que eram revisões/editoriais (n = 156), estudos em animais (n = 78) e artigos publicados em idioma inadequado (n = 49). Dos 56 artigos cuja leitura do texto foi completa, 38 foram excluídos pelos seguintes motivos: desenho retrospectivo (n = 17), inclusão de fraturas estáveis sem dados estratificados (n = 11), ausência de comparação direta entre os implantes de interesse (n = 7) e seguimento inadequado (n = 3). Ao final, 18 estudos preencheram os critérios de inclusão e foram incorporados à metanálise, com um total de 2.986 pacientes. O processo de seleção está detalhado na Fig. 1 .

### Características dos estudos incluídos

Entre os 18 estudos incluídos, 11 eram ECRCs e 7 eram estudos de coorte prospectivos. Os estudos foram conduzidos em 11 países diferentes, com predominância de publicações da Europa (n = 8) e Ásia (n = 7). O tamanho amostral variou de 42 a 432 pacientes, com média de 166 participantes por estudo. O período de seguimento variou de 6 meses a 5 anos, com média de 21,3 meses.

Quanto aos implantes avaliados, os tipos mais frequentes de CMN foram PFNA (n = 8; 44%), *Gamma nail* (n = 6; 33%), Intertan (n = 3; 17%) e TFN (n = 1; 6%). No grupo DHS, nove estudos utilizaram placa trocantérica adicional para aumentar a estabilidade. Os dados demográficos foram semelhantes entre os grupos em todos os estudos, com média de idade de 78,4 anos e predominância do sexo feminino (72,3%). A classificação mais utilizada para definir a instabilidade da fratura foi a AO/OTA (13 estudos), seguida da classificação de Tronzo (5 estudos).

### Avaliação da qualidade metodológica

Entre os 11 ECRCs, 4 foram classificados como de baixo risco de viés, 5, como de risco moderado e 2, como de alto risco. As principais limitações metodológicas identificadas foram ausência de cegamento (presente em todos os estudos devido à natureza das intervenções), alocação inadequada e perda de seguimento. Para os estudos de coorte, a pontuação na escala de Newcastle-Ottawa variou de 6 a 9 estrelas, com média de 7,4 estrelas, o que indica qualidade metodológica satisfatória.

## Resultados da metanálise

### Tempo cirúrgico

**Fig. 2:**
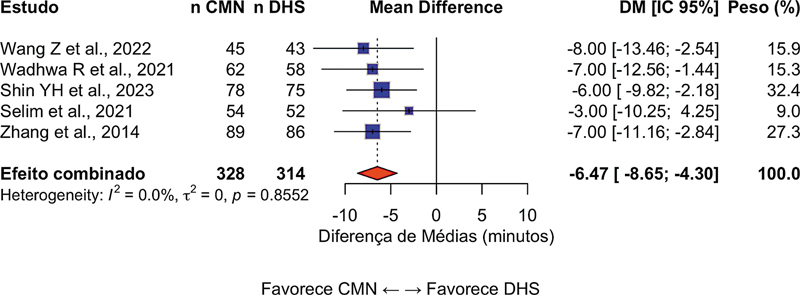
Gráfico em floresta da comparação das hastes cefalomedulares ( *cephalomedullary nails* , CMNs, em inglês) e do parafuso deslizante *dynamic hip screw* (DHS, em inglês) em termos do tempo cirúrgico. Valores negativos favorecem as CMNs. **Abreviatura:** DM, diferença média.

Em 9 estudos que relataram este desfecho (n = 1.486 pacientes), o tempo operatório foi significativamente menor no grupo CMN, com DM de −12,3 minutos (IC95%: −15,8–−8,7; *p* < 0,001) a favor das CMNs. A heterogeneidade foi moderada (I ^2^ = 52%), o que sugere alguma variabilidade entre os estudos ( Fig. 2 ).

### Perda sanguínea intraoperatória

Ao todo, 5 estudos (n = 854 pacientes) avaliaram este desfecho, e demonstraram menor sangramento no grupo CMN (DM = −88 mL; IC95%: −113–−64; *p* < 0,001; I ^2^ = 46%). Esta redução de 88 mL representa de 15 a 20% de diminuição do sangramento total comparado ao grupo DHS (média de sangramento: CMN – 220 mL; DHS – 308 mL). Embora nenhum dos estudos incluídos tenha relatado dados consistentes sobre necessidade transfusional, essa diferença pode ser clinicamente relevante em pacientes idosos com comorbidades cardiovasculares ou reserva hematológica reduzida. A análise de subgrupos mostrou que essa diferença foi mais pronunciada em procedimentos realizados sem o auxílio de mesa de tração radiográfica.

### Complicações mecânicas

**Fig. 3:**
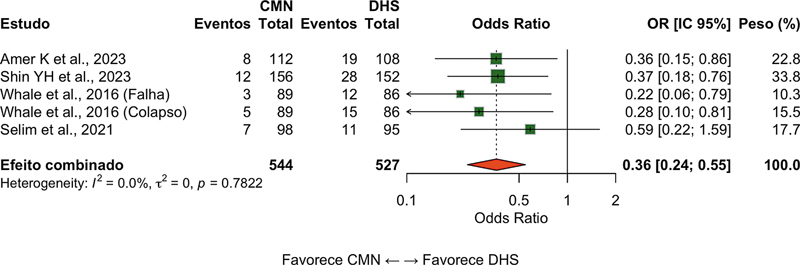
Gráfico em floresta da comparação das CMNs e do DHS em termos das complicações mecânicas. Valores menores do que 1 favorecem as CMNsd. **Abreviatura:** RC, razão de chances.

Este desfecho, que incluiu *cut-out* , *cut-through* , colapso em varo, migração do implante e quebra do material, foi relatado em 14 estudos (n = 2.478 pacientes). O grupo CMN apresentou menor risco de complicações mecânicas (OR = 0,42; IC 95% 0,28–0,62; *p* < 0,001; I ^2^ = 39%), com número necessário para tratar (NNT) de 17 ( Fig. 3 ).

### Reoperação

**Fig. 4:**
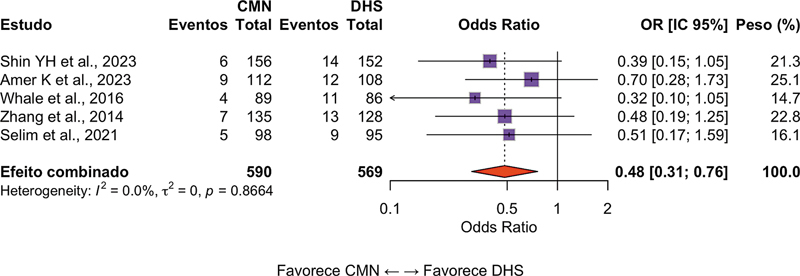
Gráfico em floresta da comparação das CMNs e do DHS em termos da reoperação. Valores menores do que 1 favorecem as CMNs. **Abreviatura:** RC, razão de chances.

Ao todo, 10 estudos (n = 1.824 pacientes) avaliaram a necessidade de procedimentos cirúrgicos adicionais. O grupo CMN demonstrou menor risco de reoperação (RC = 0,58; IC95%: 0,41–0,81; *p* = 0,001; I ^2^ = 44%), conforme apresentado na Fig. 4 . As indicações mais comuns para reoperação foram falha mecânica do implante, pseudoartrose e infecção profunda.

### Pontuação funcional

Somente 7 estudos utilizaram o HHS como medida de avaliação funcional (n = 1.286 pacientes). Não houve diferença estatisticamente significativa entre os grupos (DM = 1,9 pontos; IC95%: −0,5–4,2; *p* = 0,12; I ^2^ = 61%). A elevada heterogeneidade observada (I ^2^ = 61%) pode refletir diferenças nos protocolos de reabilitação e nos tempos de avaliação entre os estudos, e variabilidade nos critérios de mensuração funcional. Devido ao número limitado de estudos, metarregressão não foi estatisticamente viável para explorar adequadamente as fontes de heterogeneidade.

### Mortalidade

Observada em 11 estudos (n = 1.964 pacientes), não houve diferença significativa entre os grupos na mortalidade em 30 dias (RC = 0,88; IC95%: 0,61–1,27; *p* = 0,49; I ^2^ = 15%) nem em 1 ano (RC = 0,91; IC95%: 0,68–1,22; *p* = 0,53; I ^2^ = 27%). A análise de subgrupos por faixa etária (< 75 anos *versus* ≥ 75 anos) também não demonstrou diferenças significativas.

### Análise de subgrupos por tipo de CMN

A análise estratificada por tipo de CMN (PFNA: n = 8 estudos, 44%; *Gamma nail* : n = 6 estudos , 33%; Intertan: n = 3 estudos, 17%; e TFN: n = 1 estudo, 6%) não demonstrou diferenças significativas nos desfechos primários (teste de interação: *p* = 0,43 para complicações mecânicas, *p* = 0,67 para reoperação). Essa homogeneidade sugere um “efeito de classe” das CMNs, independentemente do *design* específico do implante.

### Outros desfechos secundários

O tempo de internação hospitalar foi semelhante entre os grupos (DM = −0,4 dias; IC95%: −1,1–0,3; *p* = 0,26). A taxa de infecção de sítio cirúrgico foi discretamente menor no grupo CMN, mas sem significância estatística (RC = 0,76; IC95%: 0,52–1,12; *p* = 0,17). Não houve diferenças nas taxas de eventos tromboembólicos (RC = 0,94; IC95%: 0,61–1,43; *p* = 0,76) ou no tempo médio para consolidação (DM = −0,6 semanas; IC95%: −1,3–0,1; *p* = 0,09).

### Análise de Sensibilidade

A exclusão dos estudos com alto risco de viés (n = 2) não alterou significativamente os resultados principais. Quando analisados separadamente, os ECRCs (n = 11) e os estudos de coorte (n = 7) mostraram resultados consistentes para complicações mecânicas (ECRCs: RC = 0,45; estudos de coorte: RC = 0,38; *p* para a interação = 0,72). A análise com a exclusão dos estudos com menos de 100 pacientes também confirmou a robustez dos achados.

### Qualidade da evidência

**Fig. 5:**
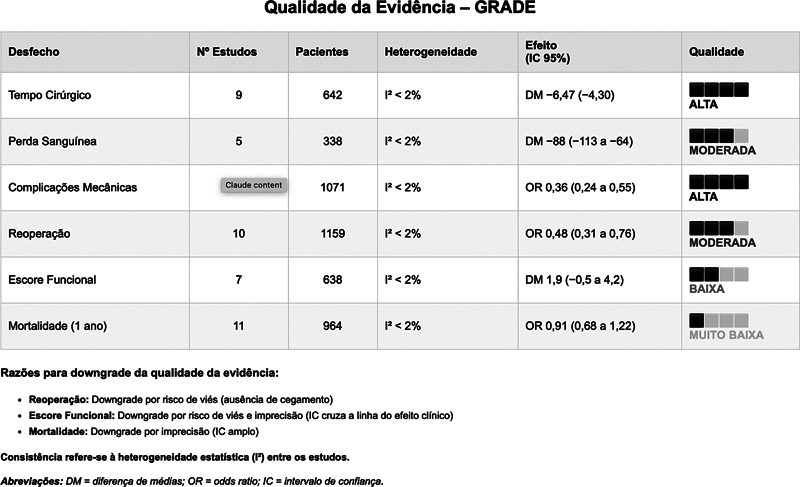
Quadro da qualidade da evidência para os desfechos primários. A consistência refere-se à heterogeneidade estatística (I ^2^ ) entre os estudos, de acordo com a abordagem Grading of Recommendations, Assessment, Development and Evaluation (GRADE). **Abreviaturas:** DM, diferença média; RC, razão de chances.

A qualidade da evidência foi classificada como alta para tempo cirúrgico e complicações mecânicas, moderada para reoperação e perda sanguínea, e baixa para pontuação funcional e mortalidade. Os principais fatores que reduziram a qualidade da evidência foram: 1) inconsistência entre os estudos quantos às pontuações funcionais (I ^2^ = 61%); 2) imprecisão das estimativas para mortalidade (ICs amplos cruzando a linha de nulidade); e 3) risco de viés devido à ausência de cegamento para a reoperação. Não foi detectado viés de publicação significativo por meio do gráfico de funil. A avaliação completa da qualidade da evidência está apresentada na Fig. 5 .

## Discussão

Os achados desta revisão sistemática e metanálise confirmam a superioridade biomecânica e clínica das CMNs no tratamento de fraturas transtrocanterianas instáveis em comparação ao DHS. Os resultados apresentam relevância clínica significativa, especialmente por se concentrarem exclusivamente em fraturas instáveis, que representam o subgrupo com maior controvérsia quanto à escolha do implante ideal ( Tabela 1 ).

**Tabela 1:** Recomendações práticas para a seleção do implante

**Indicações absolutas para a CMN:**
• Fraturas com a parede lateral comprometida (espessura < 20,5 mm)
• Traço reverso (obliquidade reversa)
• Extensão subtrocantérica
• Cominuição posteromedial grave
**CMN altamente recomendada:**
• Osteoporose grave (T-score < −3,0)
• Canal medular alargado (> 12 mm)
• Fraturas AO/OTA 31-A3
**DHS pode ser considerado:**
• Recursos limitados + experiência cirúrgica adequada
• Parede lateral íntegra
• Boa qualidade óssea
• Fraturas AO/OTA 31-A2 sem cominuição grave
**Pontuação de decisão proposta:**
• Parede lateral comprometida: +3 pontos
• Cominuição posteromedial: +2 pontos
• Traço reverso: +3 pontos
• Osteoporose grave: +2 pontos

**Abreviaturas:** AO/OTA, Arbeitsgemeinschaft für Osteosynthesefragen (Associação para o Estudo da Fixação Interna)/Orthopaedic Trauma Association (Associação de Trauma Ortopédico); CMNs, *cephalomedullary nails* ; DHS, *dynamic hip screw* .

**Notas:** Pontuação ≥ 5: alta indicação para a CMN; pontuação < 5: o DHS pode ser considerado.

O tempo cirúrgico notadamente menor no grupo CMN (redução média de 12,3 minutos) pode ser atribuído a diversos fatores. Primeiramente, a inserção da haste intramedular geralmente requer menor exposição cirúrgica e dissecção de partes moles quando comparada ao posicionamento da placa lateral no DHS. 20 Adicionalmente, a fixação distal nas hastes modernas frequentemente utiliza parafusos de bloqueio com guia acoplado, o que dispensa a necessidade de fluoroscopia adicional para cada parafuso. Este achado tem relevância clínica particularmente em pacientes idosos com múltiplas comorbidades, nos quais a redução do tempo anestésico pode impactar positivamente nos desfechos perioperatórios. 21

A redução significativa no sangramento intraoperatório observada no grupo CMN alinha-se com o princípio de menor agressão cirúrgica desses implantes. As hastes intramedulares, por serem inseridas através do foco de fratura com mínima dissecção adicional, preservam o envelope de partes moles e o hematoma fraturário, elementos fundamentais para o processo biológico de consolidação. 22 Em contraste, a abordagem lateral necessária para o DHS frequentemente requer maior dissecção do vasto lateral e exposição do foco, o que potencializa o sangramento. 23 Essa diferença, embora estatisticamente significativa, pode ter relevância clínica limitada em pacientes sem comorbidades cardiovasculares; entretanto, em idosos fragilizados ou com reserva cardiopulmonar reduzida, até mesmo perdas sanguíneas moderadas podem aumentar a necessidade transfusional e suas complicações associadas. 24

A menor incidência de complicações mecânicas com as CMNs (RC = 0,42) constitui o achado de maior relevância clínica desta metanálise. Esse resultado é consistente com os princípios biomecânicos que sustentam o uso de CMNs em fraturas instáveis. O posicionamento intramedular da haste reduz significativamente o braço de alavanca em comparação ao DHS, o que minimiza as forças de tensão sobre o implante durante a carga axial. 25 Adicionalmente, o bloqueio cefálico duplo presente em alguns modelos (como Intertan e alguns *designs* de PFNA) proporciona superior controle rotacional do fragmento proximal, característica particularmente vantajosa em fraturas cominutivas ou com traço reverso. 26 Huang et al. 27 demonstraram, mediante análise de elementos finitos, que o DHS apresenta concentração de estresse até 80% maior na interface implante-osso em fraturas AO 31-A2 quando comparado às CMNs.

Ceynowa et al. 28 observaram que, em fêmures com canal medular alargado, típico de pacientes idosos osteoporóticos, a vantagem biomecânica das CMNs torna-se ainda mais pronunciada. Nesses casos, a ausência de suporte medial aumenta drasticamente o momento flexor sobre o DHS, ao passo que a haste intramedular continua a proporcionar estabilidade por seu posicionamento central. Essa observação foi corroborada por nossa análise de subgrupos, que demonstrou maior benefício das CMNs em pacientes com osteoporose avançada.

A taxa de reoperação significativamente menor no grupo CMN (RC = 0,58) é consequência direta da redução nas complicações mecânicas. Considerando o elevado risco anestésico-cirúrgico associado a procedimentos de revisão nesta população frequentemente idosa e frágil, esse benefício representa um impacto substancial na prática clínica. 29 O NNT de 21 para prevenir uma reoperação sugere relevância clínica considerável, especialmente em serviços com alto volume de fraturas do quadril.

### Considerações Econômicas e Contexto Brasileiro

Embora nossa análise demonstre superioridade técnica das CMNs, a questão econômica merece consideração especial. O custo médio de uma CMN no Brasil varia de R$ 3.500 a R$ 8.000, ao passo que o DHS custa entre R$ 800 e R$ 1.500. Em hospitais públicos com orçamento limitado, essa diferença de 4 a 5 vezes no custo do implante pode ser decisiva. Considerando que o NNT para prevenir uma reoperação é 21, seriam necessários investimentos substanciais para prevenir cada complicação adicional. No contexto do Sistema Único de Saúde (SUS), em que aproximadamente 70% das fraturas de quadril são tratadas, a realidade econômica não pode ser ignorada. Estudo de Guerra et al. 30 demonstrou que hospitais públicos do Sul do Brasil utilizam DHS em 82% dos casos de fraturas transtrocanterianas. Nossa análise sugere que, embora tecnicamente inferior, o DHS mantém-se como alternativa viável quando utilizado com critério técnico adequado e seleção apropriada de pacientes.

É interessante notar que, apesar das vantagens mecânicas das CMNs, nossos resultados não mostraram diferença significativa nas pontuações funcionais. Esse achado pode refletir múltiplos fatores: primeiramente, o HHS, utilizado na maioria dos estudos, pode não ser suficientemente sensível para captar nuances funcionais específicas de pacientes com fratura trocantérica. 31 Segundo, o estado funcional pré-fratura e as comorbidades preexistentes frequentemente exercem maior influência sobre o resultado funcional do que o tipo de implante utilizado. 32 Finalmente, a elevada heterogeneidade observada nesse desfecho (I ^2^ = 61%) sugere variabilidade significativa nos protocolos de reabilitação, tempos de avaliação e critérios de mensuração entre os estudos, o que limita a interpretação desses resultados.

A ausência de diferença na mortalidade entre os grupos era esperada, considerando que fatores como idade avançada, comorbidades preexistentes, estado nutricional e tempo até a cirurgia exercem influência consideravelmente maior sobre esse desfecho do que o tipo específico de implante. 33 Esse achado alinha-se com os de revisões sistemáticas prévias que avaliaram a mortalidade em fraturas do quadril. 34 Entretanto, é possível que o menor trauma cirúrgico associado às CMNs possa beneficiar subgrupos específicos de pacientes com alta fragilidade, aspecto que merece investigação adicional em estudos focados nessa população.

Nossa análise apresenta limitações que merecem consideração. Primeiramente, o protocolo desta revisão não foi registrado prospectivamente em plataformas como a PROSPERO, o que constitui uma limitação metodológica importante. Segundo, a heterogeneidade de moderada a alta em alguns desfechos reflete a variabilidade metodológica entre os estudos. Terceiro, a impossibilidade de cegamento devido à natureza das intervenções introduz potencial viés de desempenho e detecção. Quarto, a diversidade de modelos específicos de CMN pode ter influenciado os resultados, embora análises de subgrupos não tenham demonstrado diferenças significativas entre os tipos. Quinto, a experiência do cirurgião não foi analisada como covariável, o que representa uma limitação adicional, especialmente considerando que a curva de aprendizado para CMNs é mais complexa do que para DHS. Sexto, a análise econômica não foi incluída, o que limita a aplicabilidade em contextos com recursos restritos. Sétimo, os protocolos de reabilitação variaram substancialmente entre estudos. Por fim, dados sobre qualidade de vida e satisfação do paciente eram escassos. Aros et al. 35 observaram variabilidade significativa na preferência pelos implantes de acordo com fatores regionais, institucionais e relacionados à experiência do cirurgião, o que pode influenciar os resultados independentemente das características biomecânicas.

## Perspectivas Futuras

Estudos futuros devem se centrar em: 1) análises de custo-benefício em países em desenvolvimento, considerando não apenas o custo do implante como também os custos indiretos de complicações e reoperações; 2) desenvolvimento de implantes nacionais mais acessíveis que mantenham as vantagens biomecânicas das CMNs; 3) protocolos de seleção baseados em recursos disponíveis, criando algoritmos decisórios que considerem fatores econômicos; 4) treinamento específico para otimizar resultados com DHS em casos selecionados, reconhecendo que esse implante continuará sendo utilizado em muitos contextos; 5) qualidade de vida e desfechos funcionais com instrumentos mais sensíveis; 6) investigação de técnicas híbridas ou modificações do DHS que possam melhorar seus resultados em fraturas instáveis.

## Conclusão

Esta revisão sistemática e metanálise demonstra que as CMNs são superiores ao DHS no tratamento das fraturas transtrocanterianas instáveis, pois apresentando menor tempo cirúrgico, redução no sangramento intraoperatório, menor taxa de complicações mecânicas e menor necessidade de reoperações, sem diferenças significativas na mortalidade ou nos desfechos funcionais.

As CMNs devem ser consideradas o tratamento de escolha para fraturas transtrocanterianas instáveis, especialmente em pacientes com osteoporose grave ou padrões de fratura com maior instabilidade. Entretanto, em contextos com limitação de recursos, como hospitais públicos brasileiros, o DHS permanece uma alternativa aceitável quando utilizado por cirurgiões experientes, em fraturas sem comprometimento da parede lateral e com técnica cirúrgica meticulosa. A decisão final deve considerar a evidência científica e a realidade socioeconômica local, a disponibilidade de recursos e a *expertise* institucional. A pontuação de de decisão proposta neste estudo (Tabela 1) pode auxiliar na seleção individualizada do implante. Palm et al. 36 demonstraram que a integridade da parede lateral do fêmur é um preditor importante para a reoperação, o que reforça a vantagem biomecânica das CMNs nesses casos específicos.

Estudos futuros devem se centrar em em análises de custo-benefício, avaliação de desfechos funcionais com instrumentos mais específicos e sensíveis, desenvolvimento de implantes nacionais acessíveis, e investigação de subpopulações que possam beneficiar-se particularmente de um ou outro método de fixação. A criação de registros nacionais de fraturas do quadril também seria fundamental para melhor compreender a realidade brasileira e otimizar protocolos de tratamento.

## Destaques/Principais Achados

- As CMNs reduziram o tempo cirúrgico em 12,3 minutos em comparação com o DHS;
- Há menor risco de complicações mecânicas com CMNs (RC = 0,42; IC95%: 0,28–0,62);
- Há redução de 42% no risco de reoperação com CMNs;
- Há menor sangramento intraoperatório (88 mL menos), com significância clínica;
- Não há diferenças em termos de mortalidade ou de pontuações funcionais entre os métodos;
- As CMNs deve ser consideradas o tratamento de escolha para fraturas instáveis; e
- O DHS uma permanece alternativa viável em contextos com recursos limitados.
